# The inhibitory role of stigmasterol on tumor growth by inducing apoptosis in Balb/c mouse with spontaneous breast tumor (SMMT)

**DOI:** 10.1186/s40360-022-00578-2

**Published:** 2022-06-20

**Authors:** Mandana AmeliMojarad, Melika AmeliMojarad, Alireza Pourmahdian

**Affiliations:** Department of Medical Biotechnology, School of Advanced Technologies in Medicine, Tehran University of Medical Sciences, Tehran, Iran

**Keywords:** Stigmasterol, Apoptosis, BCL-XL, BCL-2, Breast cancer

## Abstract

**Background:**

Breast cancer is one of the most common types of cancer in women worldwide. Anti-apoptotic activity of cancer cells is considered the main reason for drug resistance in BC which reduces the 5-year survival rate of patients and is still considered the main obstacle for cancer therapy. Stigmasterol (SS) is natural phytosterols compound in the plant which has been proved to play an important role to lower cholesterol and inducing anti-inflammatory, and anticancer properties.

**Methods:**

In this, study, we aimed to evaluate the effect of SS on the expression of anti-apoptotic genes (Bcl-2 and BCL-XL), and also evaluate its effects on cell apoptosis and cell viability using MCF-7 cell line as well as evaluating its effect on tumor growth of spontaneous breast tumor (SMMT) in vivo.

**Result:**

SS significantly decreased the expression of Bcl-2 and BCL-XL genes (*P < 0.05), induced apoptosis, and reduced cell proliferation in MCF-7 cell lines. Our in vivo study also indicated that SS could inhibit tumor size after treatment with (0, 10, 20 µM) compared to the normal control.

**Conclusion:**

SS can be suggested as a potential agent in BC cancer treatment or as an adjuvant based on its ability to decrease the expression of Bcl-2 and BCL-XL genes and induce apoptosis.

## Introduction

Breast cancer (BC) has become one of the leading causes of death among women in the world; BC is considered the second deadliest cancer after lung cancer [1, 2]. Currently, several treatments exist for BC patients, including surgery, radiation therapy, chemotherapy, gene therapy, and combination therapy [3]. However, the most relevant way to fight cancer is chemotherapy, which induces many side effects such as fatigue, nausea, and hair loss, that are affecting the patient’s quality of life. Therefore, it is necessary to find an alternative anti-cancer strategy with the least side effects for patients [4, 5]. Phytochemicals with their anti-cancer properties have been recently considered a new natural herbal drug in cancer treatments [6]. These compounds include phytosterols which are also known as plant sterols [7]. Therefore, in this study, we aimed to explore the effects of plant organic sterol called stigmasterol (SS) as a strategy for the development of novel anticancer drugs with lower toxicity in breast cancer. We used the MCF-7 cell line as an investigative tool based on its easy adoption in laboratories worldwide [8]. The human breast cancer cell line MCF-7 can be easily cultured in simple standard media and is an ideal cell line to study the interaction between a drug and a cancer cell to evaluate the inhibitory effects of selective drugs on the proliferation of a cancer cell in vitro [9] New research has shown that phytosterols can reduce and prevent the risk of cancer by increasing the activity of antioxidant enzymes [8, 10]. Among the members of the phytosterol’s family, SS with its great cytotoxic effects has emerged as a new plant sterol with important pro-apoptotic and antioxidant activity in different cancers including breast cancer [11]. However, the antitumor mechanism of SS has not been fully elucidated in breast cancer treatment yet. Therefore, understanding its mechanism in inducing apoptosis and inhibiting the cell growth in cancer cells can open a new horizon in the design of drugs and new therapies [12]. In this study, we decided to investigate the antitumor effects of SS on breast tumors of spontaneous mouse mammary tumor (SMMT) mice models injected with SS, as well as evaluate its effect on tumor size, the anti-apoptotic genes B-cell lymphoma-extra-large (Bcl-xl) and B-cell lymphoma 2(Bcl2) and apoptosis.

## Materials and methods

### Materials

All cell lines were purchased from Pasteur (Tehran, IRAN). fetal bovine serum (FBS) was obtained from (GIBCO, USA), RPMI 1640 medium (Gibco, Carlsbad, CA, USA), cDNA Synthesis Kit (Thermo Scientific), Annexin V-FITC binding buffer from (Bio Legend, San Diego, USA), SYBR® qPCR Mix from (Toyobo, Osaka, Japan).

### Reagents source

Stigmasterol (purity ≥ 98%) dried powder was purchased from (Exim Pharm Co) Stigmasterol was dissolved in the minimum amount of pure ethanol (Merck, USA) and was mixed with PBS. The final PBS / ethanol ratio was equal to (1: 2). 9 SMMT mice and normal Balb / C mice were obtained from the Iranian Pasteur Institute. All animals in the experiments are approved by the Ethics Committee of Medical University of Tehran and the process was in accordance with animal rules. The study was approved by the Faculty of Science, Medical University Animal of Tehran, Care and Use Committee.

### Cell culture and treatment

Human breast cancer cell line MCF-7, and normal cell line (MCF10A) were purchased from the Institute Pasteur of Iran, (Tehran, Iran). RPMI 1640 medium (Gibco, Carlsbad, CA, USA) containing 10% fetal bovine serum (FBS) (GIBCO, USA), was used for cell culture at 37 °C and a 5% CO2. Afterward, MCF-7 were treated with different doses of SS (0, 10, 20 µM) for 72 h.

### RNA extraction and real-time PCR

RNA was extracted from tumor tissue of stigmasterol-treated Balb / c mice using a Qiagen kit. After extracting the RNA quality was measured by nano drops (Thermo Scientific). Next, we synthesize cDNA by using the cDNA Synthesis Kit (Thermo Scientific) for performing the qRT-PCR using (THUNDERBIRD SYBR® qPCR Mix) (Toyobo, Osaka, Japan). GAPDH was used for normalizing the expression and data were measured by the 2^−ΔΔCt^ method. Table 1 displayed the primers for this research.

**Table 1:** Primers sequences

Gene	UniProt ID	Forward	Reverse
BCL2	P10415	**ATCGCCCTGTGGATGACTGAGT**	**GCCAGGAGAAATCAAACAGAGGC**
BCL-XL	Q07817	**GCCACTTACCTGAATGACCACC**	**AACCAGCGGTTGAAGCGTTCCT**
GAPDH	P04406	**GTCTCCTCTGACTTCAACAGCG**	**ACCACCCTGTTGCTGTAGCCAA**

### Cell viability assay

The cell-counting kit-8 (CCK-8) was used to evaluate the effect of SS on cells proliferation and viability based on the manufacturer’s instruction. 0.2 × 10^4^ density of MCF-7 cells were seeded in 96-well plates and incubated in the standard condition of 37 °C and 5% CO2 atmosphere followed by different SS concentrations (0, 10, 20, µM) for 24, 48, and 72 h CCK-8 solution was added to each well at a final concentration of 10%. Then, the optical density (OD) was measured at 450 nm using Perkin Elmer’s EnSpire Multi-Label Plate Reader (Santa Clara, USA). All results were received from three independent experiments [12].

### Apoptosis assay

To analyze the effect of SS on MCF-7, early and late apoptotic we used Annexin V-FITC/Propidium Iodide assay apoptosis detection kit. In Brief after treatments with different SS concentrations (0,10, 20, µM), cultured cells were collected and centrifugation, the cells suspended in Annexin V-FITC binding buffer (BioLegend, San Diego, USA), and dyed with FITC Annexin V and PI in the dark. The total apoptotic rate was calculated by combining early and late apoptotic cells. Using flow cytometry and cell quest software (BD Bioscience, Franklin Lakes, NJ, USA).

### Treatment of SMMT tumor mice with stigmasterol

SMMT is an invasive duct carcinoma that develops spontaneously in female Balb / c mice (20). When the tumor size reached 1500 mm3, the first group received 10 µM SS, the second group received 20 µM cyclophosphamide, and the third group (control) received ethanol solution diluted with solution PBS (GIBCO, USA) (1: 2). Tumor volume in each group was measured using a digital caliper for 30 days and calculated with the following formula: V = π / 6 × LW2 (π: 3.14, V: volume, L: length, and W: width).

### Statistical analysis of data

Data were analyzed by Graph pad prism8, and spss16. The one-way ANOVA and t-test were mainly used to analyze the data. Results are reported as (mean ± SD). Differences were considered significant when the *p*-value was 0 < 0.05.

## Results

### The effect of stigmasterol on the expression of BCL-XL and Bcl-2 gene in MCF-7 cells

**Fig. 1:**
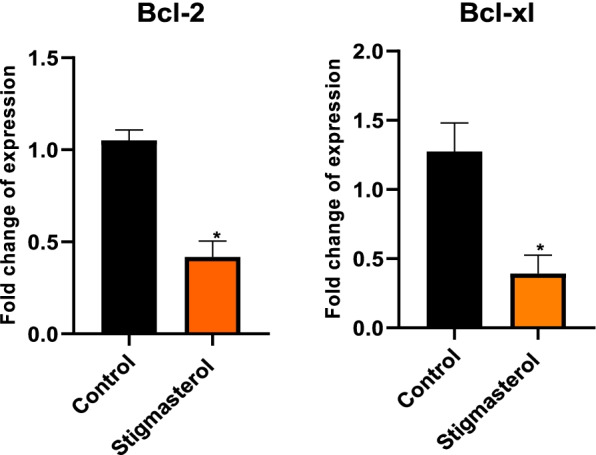
Gene expression levels of Bcl-2 and Bcl-xl after treatment with 20 µM of SS, in MCF-7 and MCF10A cell line as the control. Data are represented as mean ± SD (* *P* < 0.05)

To investigate the SS mechanism of action we first analyze the changes in the expression levels of anti-apoptotic genes such as Bcl-xl and Bcl2 genes, which are the member of the Bcl2 family proteins, this family play a key regulatory role in apoptosis by suppressing cell death by preventing the pore formation and release of cytochrome-c. Our result proposed that after treating the MCF-7 cell line with 20 (µM) of SS the expression levels of selected genes were significant decrees compared to the normal control group (MCF-10A) (*p* < 0.05) (Fig. 1).

### Effect of SS on cell proliferation

**Fig. 2:**
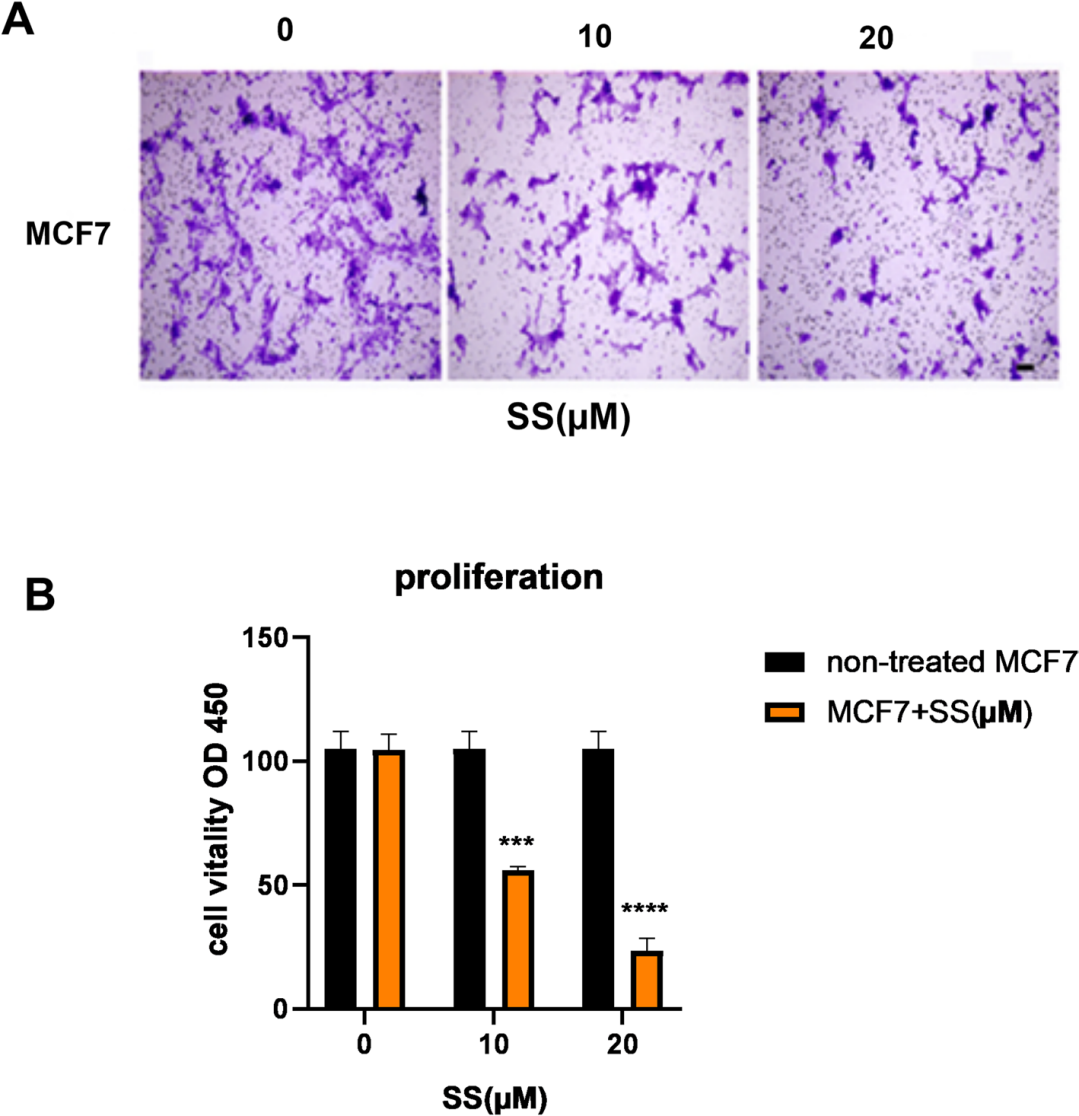
The inhibitory role of Stigmasterol (SS) on cell viability and proliferation of BC cells, the Survival rate of MCF-7 enhanced in the response to increased levels of SS. The obtained results are expressed as a percentage (A) and (B) which showed a dose and time-dependent manner. Data are presented as mean ± SD of triplicate experiments that were repeated at least three times

To evaluate the effects of SS on breast cell proliferation and viability of cancer cells, selected concentrations (0, 10, 20, µM) of SS were used to treat MCF-7 cells for (24, 48, and 72 h) by CCK-8 assay (Fig. 2A). Based on the results SS could significantly inhibit cells proliferation in a dose and time-dependent manner (Fig. 2B).

### Changes in apoptosis after treatment of cells with SS

**Fig. 3:**
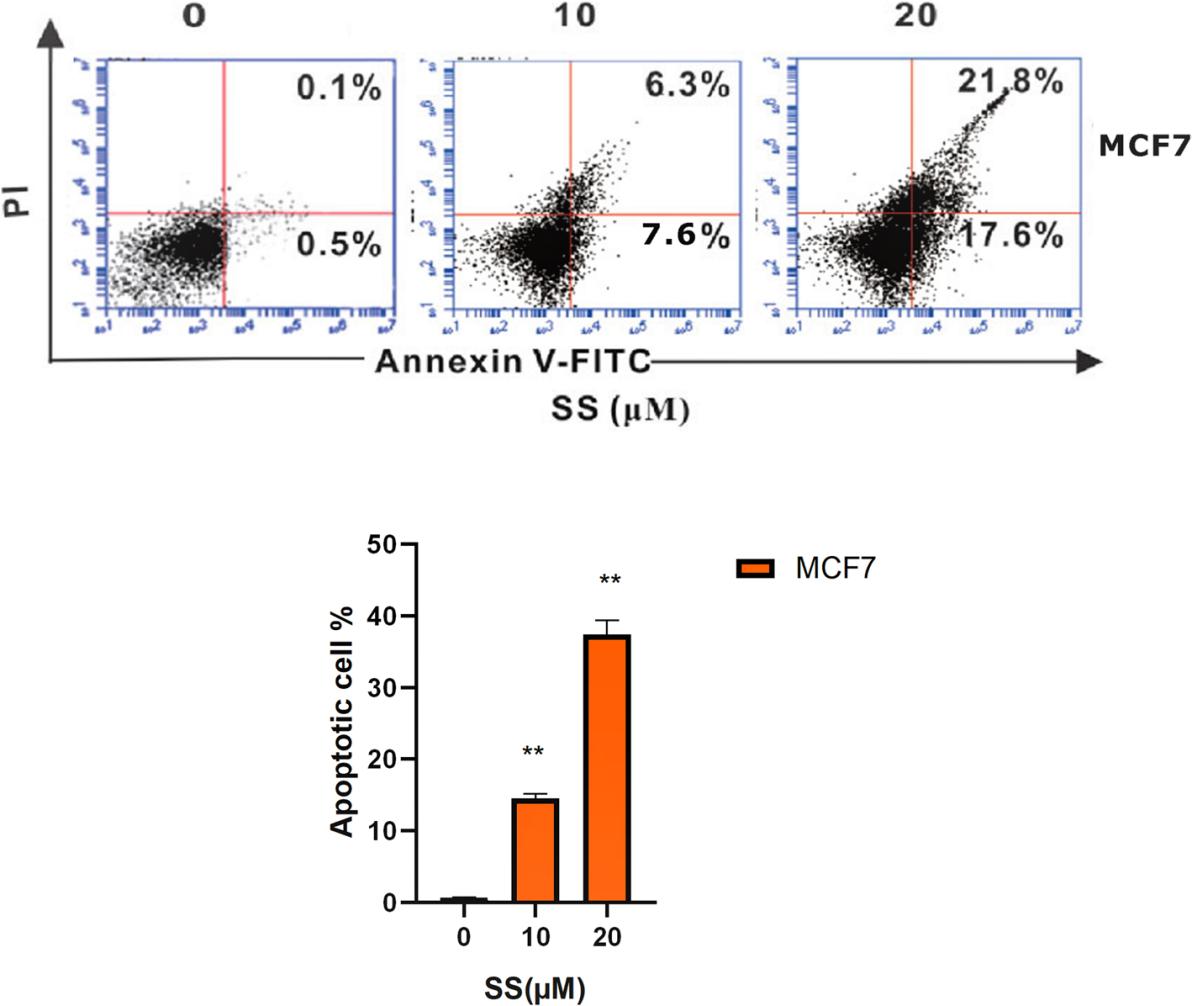
Flow cytometry for detection of apoptosis in the MCF-7 cells treated with SS. The cells were treated with mentioned concentrations and harvested after 72 h for double staining (Annexin V/PI)

Next, we calculated apoptosis assay after treatments of MCF-7 cell lines with a selected dosage of SS (0/10/20 µM) and analyzed the early and late stages of apoptosis (lower right side) and (upper right side) respectively, flow cytometry was performed by using Annexin V/PI for staining. Data showed that the percentage of apoptotic cells was increased with the increase of SS concentration and had the paralleled effects on cell death shown in (Fig. 3).

### The effect of stigmasterol on tumor size in vivo

**Fig. 4:**
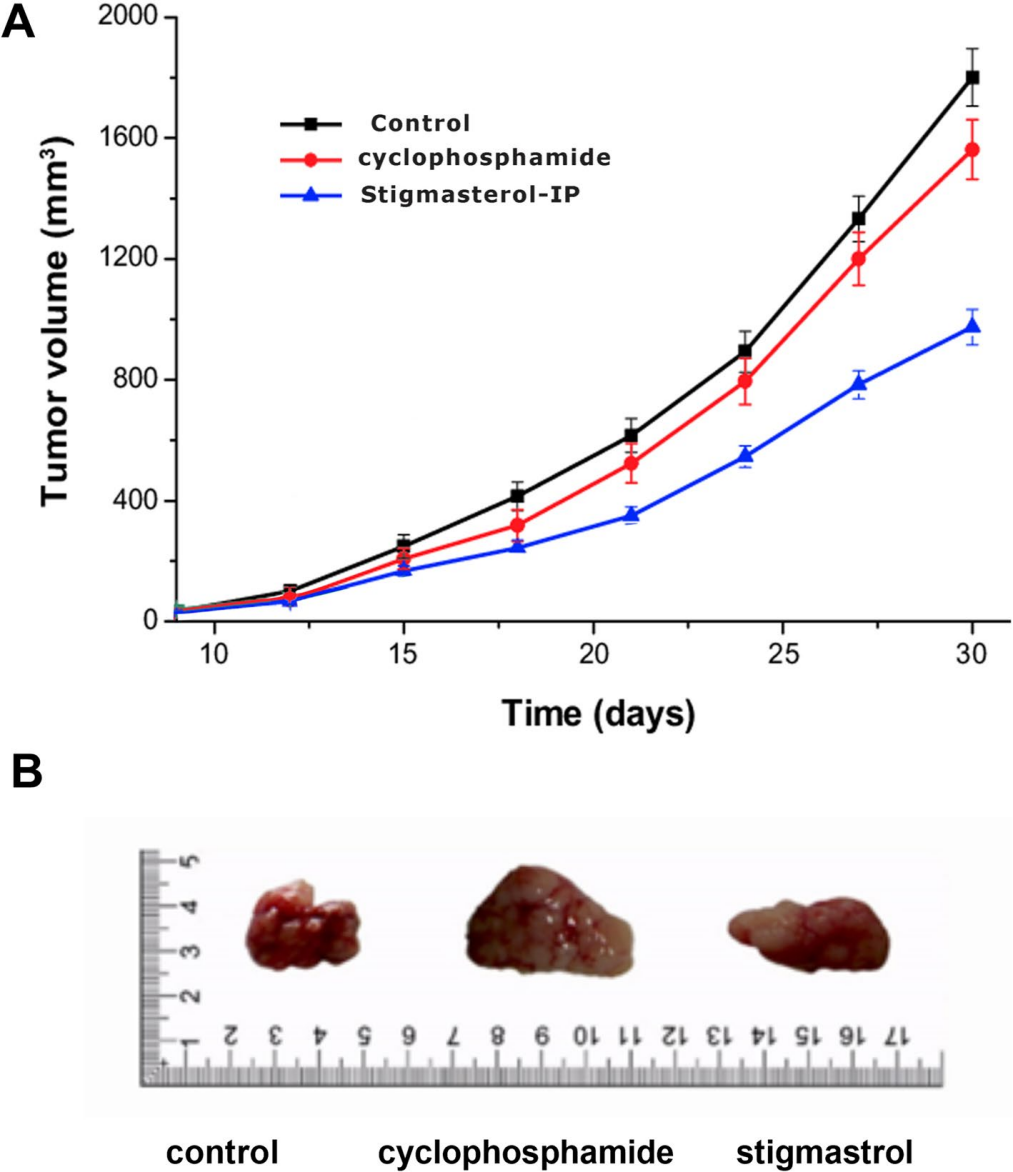
Inhibition of tumor size by Stigmasterol (**A**) Comparison of tumor diameter in Balb/c mouse after treatment, with SS compared to cyclophosphamide as the positive control (**B)** representee tumor photograph from each group

To further examine the role of SS in vivo, we inoculated SS into BALB/c-nude mice via intraperitoneal injection of SS and determined the tumor-related parameters. In this study, 9 Balb / c mice were used. The first group received a dose of 20 µM SS, the second group received a dose of 10 µM cyclophosphamide, and the third group (control) received a solution of ethanol diluted with phosphate buffer (PBS) (1: 2) via intraperitoneal (IP) injection and tumor volume was measured daily. The results showed that SS significantly reduced the tumor growth compared to the control group after 30 days of treatments (Fig. 4), these results suggested that SS could effectively inhibit growth in vivo.

## Discussion

Since tumor cells need to inhibit apoptosis for their abnormal growth, cancer cells have mediated different mechanisms to resist apoptosis and proliferation [13]. One of these mechanisms is based on over expression of various genes involved in anti-apoptosis processes, including members of the Bcl-2 family such as BCl 2, Bcl-XL, BCL-W, and MCl-1 which are elevated in a variety of cancers, including breast cancer and inhibit cell death in tumors [14–16]. Studies have indicated a direct relationship between the cancer progression and the expression of anti-apoptotic genes of the Bcl-2 family, Also over expression of Bcl2 can lead to cancer resistance in a wide range of cancers [17]. Therefore, strategies to inhibit Bcl2 can activate the apoptotic process in tumor cells [18]. As mentioned above, discovering a potential molecule to inhibit Bcl2 may open a new door to cancer treatment.Stigmasterol (SS) is a member of phytosterols which can inhibit the progression of different cancers by decreasing the tumors' viability and promoting apoptosis [12, 19]. Other pharmacological activities of SS, including anti-inflammatory, chemo sensitivity and antidiabetic, have been reported recently [20, 21]. The anticancer effects of SS were detected in various physiological processes and cancers previously for example in the skin cancer SS via suppressing the VEGFR-2 and TNF-alpha can increase lipid peroxide levels [22]. Or in prostate cancer SS by inducing p53 protein expression can suppress cancer cells developments [23], and induce apoptosis by increasing DNA damage in HepG2 cells [24], there are different studies about the inducing effects of SS on mitochondrial apoptosis signaling pathway which can be related to caspase-8 and -9 up regulation in hepatoma, or up regulation cytochrome c, BAK, and BAX and cleaved caspase-3 and 9 in ovarian cancer cell lines [23, 24]. Previous findings also illustrated the apoptosis activation role of SS via inhibiting the Akt/mTOR pathway in gastric cancer (GC) and suggesting the potential anticancer effect of SS in GC treatment [12]. SS is also an unsaturated phytosterol that is used in the biosynthesis of various hormones, such as estrogens, progesterone, androgens, and corticoids. SS can also use as an intermediate precursor of vitamin D3 [25]. Therefore, in this study, we aimed to evaluate the antitumor effects of SS on the MCF-7 cell line a human breast cancer cell line with estrogen, progesterone, and glucocorticoid receptors and spontaneous mouse mammary tumor (SMMT) for the first time. According to our observed results, the expression of selected anti-apoptotic genes (BCL-XL BCL2) were both significantly reduced after treatments with SS, our next results also indicate the SS's potential role in inducing the apoptosis and decreasing cell viability in MCF-7 cells. We also evaluate the inhibitory effects of SS on SMMT tumor volume of Balb /c mice, the significant decrease in tumor size was observed in the SS treated group compared with the control group after 30 days of treatment suggesting the potential role of SS for tumor treatments.

## Conclusion

Based on our result SS can be strongly induced the apoptotic and can be considered a potential agent in breast cancer treatment. However, more research is needed on the mechanism of action of this phytosterols compound to find a more effective strategy in different dosages and cell lines for designing a better therapy for the prevention of breast cancer.

## Data Availability

The datasets generated and/or analyzed during the current study are available from the corresponding author on reasonable request.

## References

[1] Mojarad MA, Mojarad MA, Noourbakhsh M (2022). Impact of Vitamin D on Expression of SIRT7 and CYP24A1 in Human Breast Cancer Cells. Res Vet Sci Med.

[2] Mendes PMV, Bezerra DLC, dos Santos LR, de Oliveira SR, de Sousa Melo SR, Morais JBS (2018). Magnesium in Breast Cancer: What Is Its Influence on the Progression of This Disease?. Biol Trace Elem Res.

[3] Bray F (2018). Global cancer statistics 2018: GLOBOCAN estimates of incidence and mortality worldwide for 36 cancers in 185 countries. CA Cancer J Clin.

[4] Latruffe N (2017). Natural Products and Inflammation. Mol.

[5] Mojarad MA, Mojarad MA, Wang J, Noourbakhsh M (2022). Anti-inflammation and anti-cancer effects of Naringenin combination with Artemisinins in human lung cancer cells. Gene Reports..

[6] Mojarad MA, Mojarad MA, Pourmahdian A (2021). Long Non-coding RNA snaR Promotes Proliferation in EGFR Wild Type Non-Small Cell Lung Cancer Cells. Int J Mol Cell Med.

[7] Liu L, Liu D, Xiang C, Dai W, Li B, Zhang M (2020). Sesquiterpene lactones from Artemisia austroyunnanensis suppresses ROS production and reduces cytokines, iNOS and COX-2 levels via NF-KB pathway in vitro. Nat Prod Res.

[8] Woyengo TA, Ramprasath VR, Jones PJH (2009). Anticancer effects of phytosterols. Eur J Clin Nutr.

[9] Juhasz K, Lipp AM, Nimmervoll B, Sonnleitner A, Hesse J, Haselgruebler T (2013). The complex function of hsp70 in metastatic cancer. Cancers (Basel).

[10] García-Llatas G, Rodríguez-Estrada MT (2011). Current and new insights on phytosterol oxides in plant sterol-enriched food. Chem Phys Lipid.

[11] Carter BA, Taylor OA, Prendergast DR, Zimmerman TL, Von Furstenberg R, Moore DD (2007). Stigmasterol, a Soy Lipid-Derived Phytosterol, Is an Antagonist of the Bile Acid Nuclear Receptor FXR. Pediatr Res.

[12] Zhao H, Zhang X, Wang M, Lin Y, Zhou S (2021). Stigmasterol Simultaneously Induces Apoptosis and Protective Autophagy by Inhibiting Akt/mTOR Pathway in Gastric Cancer Cells. Front Oncol.

[13] AmeliMojarad M, AmeliMojarad M, Pourmahdian A (2022). Circular RNA circ_0051620 sponges miR-338-3p and regulates ADAM17 to promote the gastric cancer progression. Pathol Res Pract.

[14] Letai A. Pharmacological manipulation of Bcl-2 family members to control cell death. J Clin Invest. 2005;115(10):2648-55. 10.1172/JCI26250. 10.1172/JCI26250 PMC1236690 16200198

[15] Lucantoni F, Salvucci M, Düssmann H, Lindner AU, Lambrechts D, Prehn J. BCL(X)L and BCL2 increase the metabolic fitness of breast cancer cells: a single-cell imaging study. Cell Death Differ. 2021;28(5):1512–31. 10.1038/s41418-020-00683-x. 10.1038/s41418-020-00683-x PMC8166899 33328572

[16] Campbell KJ, Dhayade S, Ferrari N, Sims AH, Johnson E, Mason SM (2018). MCL-1 is a prognostic indicator and drug target in breast cancer article. Cell Death Dis.

[17] Matsumoto A, Isomoto H, Nakayama M, Hisatsune J, Nishi Y, Nakashima Y (2011). Helicobacter pylori VacA reduces the cellular expression of STAT3 and pro-survival Bcl-2 family proteins, Bcl-2 and Bcl-X L, leading to apoptosis in gastric epithelial cells. Dig Dis Sci.

[18] Vogler M (2012). BCL2A1: the underdog in the BCL2 family. Cell Death Differ.

[19] Bae H, Song G, Lim W (2020). Stigmasterol causes ovarian cancer cell apoptosis by inducing endoplasmic reticulum and mitochondrial dysfunction. Pharmaceutics.

[20] Liao H, Zhu D, Bai M, Chen H, Yan S, Yu J (2020). Stigmasterol sensitizes endometrial cancer cells to chemotherapy by repressing Nrf2 signal pathway. Cancer Cell Int.

[21] Poulose N, Sajayan A, Ravindran A, Chandran A, Priyadharshini GB, Selvin J (2021). Anti-diabetic Potential of a Stigmasterol From the Seaweed Gelidium spinosum and Its Application in the Formulation of Nanoemulsion Conjugate for the Development of Functional Biscuits. Front Nutr.

[22] Ali H, Dixit S, Ali D, Alqahtani SM, Alkahtani S, Alarifi S (2015). Isolation and evaluation of anticancer efficacy of stigmasterol in a mouse model of DMBA-induced skin carcinoma. Drug Des Devel Ther.

[23] Borner MM, Brousset P, Pfanner-Meyer B, Bacchi M, Vonlanthen S, Hotz MA (1999). Expression of apoptosis regulatory proteins of the Bcl-2 family and p53 in primary resected non small-cell lung cancer. Br J Cancer.

[24] Kim YS, Li XF, Kang KH, Ryu BM, Kim SK (2014). Stigmasterol isolated from marine microalgae Navicula incerta induces apoptosis in human hepatoma HepG2 cells. BMB Rep.

[25] Sundararaman P, Djerassi C (1977). A Convenient Synthesis of Progesterone from Stigmasterol. J Org Chem.

